# Aptamer based point of care diagnostic for the detection of food allergens

**DOI:** 10.1038/s41598-022-05265-0

**Published:** 2022-01-25

**Authors:** Sarah Stidham, Valerie Villareal, Vasant Chellappa, Lucas Yoder, Olivia Alley, Wayne Shreffler, Jonathan Spergel, David Fleischer, Hugh Sampson, Adi Gilboa-Geffen

**Affiliations:** 1 DOTS Technology Corp, Natick, USA; 2 Massachusetts General Hospital, Boston, USA; 3 Children’s Hospital of Philadelphia, Philadelphia, USA; 4 Children’s Hospital Colorado, Aurora, USA; 5 Mount Sinai, New York, USA

**Keywords:** DNA, Assay systems

## Abstract

Aptamers, due to their small size, strong target affinity, and ease of chemical modification, are ideally suited for molecular detection technologies. Here, we describe successful use of aptamer technology in a consumer device for the detection of peanut antigen in food. The novel aptamer-based protein detection method is robust across a wide variety of food matrices and sensitive to peanut protein at concentrations as low as 12.5 ppm (37.5 µg peanut protein in the sample). Integration of the assay into a sensitive, stable, and consumer friendly portable device will empower users to easily and quickly assess the presence of peanut allergens in foods before eating. With many food reactions occurring outside the home, the type of technology described here has significant potential to improve lives for children and families.

## Introduction

Aptamers are oligonucleotides capable of high-affinity binding to target molecules^1^. Since development of the in vitro systematic evolution of ligands by exponential enrichment (SELEX) selection process in 1990, aptamers have been designed to selectively bind diverse targets, including RNA, DNA, and other small molecules and compounds^2–4^. These use cases have supported their development as valuable tools for fundamental research, therapeutic applications, and as sensors in molecular diagnostic devices^3,5^. Due to their high affinity, small size, and ease of chemical modification, aptamers have been suggested as a superior reagent for molecular target recognition. They have also gained traction in several clinical applications, with the first aptamer-based therapeutic gaining FDA approval in 2004^6^.

The conventional method of detecting antigen is through antibody recognition. Approaches, such as enzyme linked immunosorbent assays (ELISA), immunoblots, visualization with microscopy, etc., that indirectly identify targets via primary and secondary antibody recognition have been used extensively throughout basic research. For a sensor technology, however, aptamers, which can exhibit high affinity and specificity comparable to monoclonal antibodies^5^, may have advantages. Accordingly, there have been several attempts to create an aptamer-based sensor for the detection of peanut protein, specifically Ara h 1. These sensors have featured graphene oxide^7^, origami nano-aptasensors^8^, or fiber optics surface plasmon resonance biosensing technologies^9^. Universally, these have been stand-alone sensors, which do not have incorporated sample preparation steps, such as homogenizing of the food or extraction of the peanut protein. This means substantial sample prep, which can take more than 30 min to perform and require kits and laboratory equipment to perform, is necessary. These factors preclude the use of these sensors by consumers. Moreover, limited testing of the sensors, typically only with purified Ara h 1 in buffer and/or a single food, has prevented full analysis of the robustness of these sensors to complex food matrices and additives^7–10^.

Given that many food reactions occur upon consumption of food outside of the home^11–13^, a sensor capable of detecting the presence of peanut protein in foods in a portable, stable, and robust format is needed^14^. Here, for the first time, we describe an end-to-end solution, inclusive of sample prep and antigen detection, that takes approximately 3 min to perform and is integrated into a portable consumer-friendly device format. Extensive testing demonstrates the sensor is capable of sensitive detection of peanut protein in over 50 foods.

There are 32 million^15,16^ people with food allergies in the United States, and allergic reactions lead to approximately 200,000 emergency department visits^17^ and 200 deaths each year^18^. Strict avoidance of allergens in the diet is the sole treatment for food allergies, but due to the ubiquity of the most common allergens in the food chain the risk of accidental exposure is high^19–21^. Food allergy management requires individuals and caregivers to continuously manage exposure to allergens^22^, and food prepared and consumed outside the home can be in question^22^ due to lack of awareness and knowledge about food allergies among restaurant workers^23^. Due to these, children and families experience psychological, social, and economic burdens and caregivers of children with food allergies often experience diminished quality of life, anxiety, and frustration over lack of food allergy awareness^24^. The proposed assay described here can empower consumers to easily and quickly assess the presence of allergens in foods before eating to help people with food allergies and intolerances manage their daily life.

## Results

### Aptamer selection

Five aptamers were initially chosen (P1-16, P1-10, PT-31, P2-8, and P2-18), based on a peanut-targeted SELEX pool^1,25^. To develop an aptamer-based assay with an optical readout, the aptamers were conjugated with a Texas Red (TR) fluorophore on the 5′ end.

**Figure 1:**
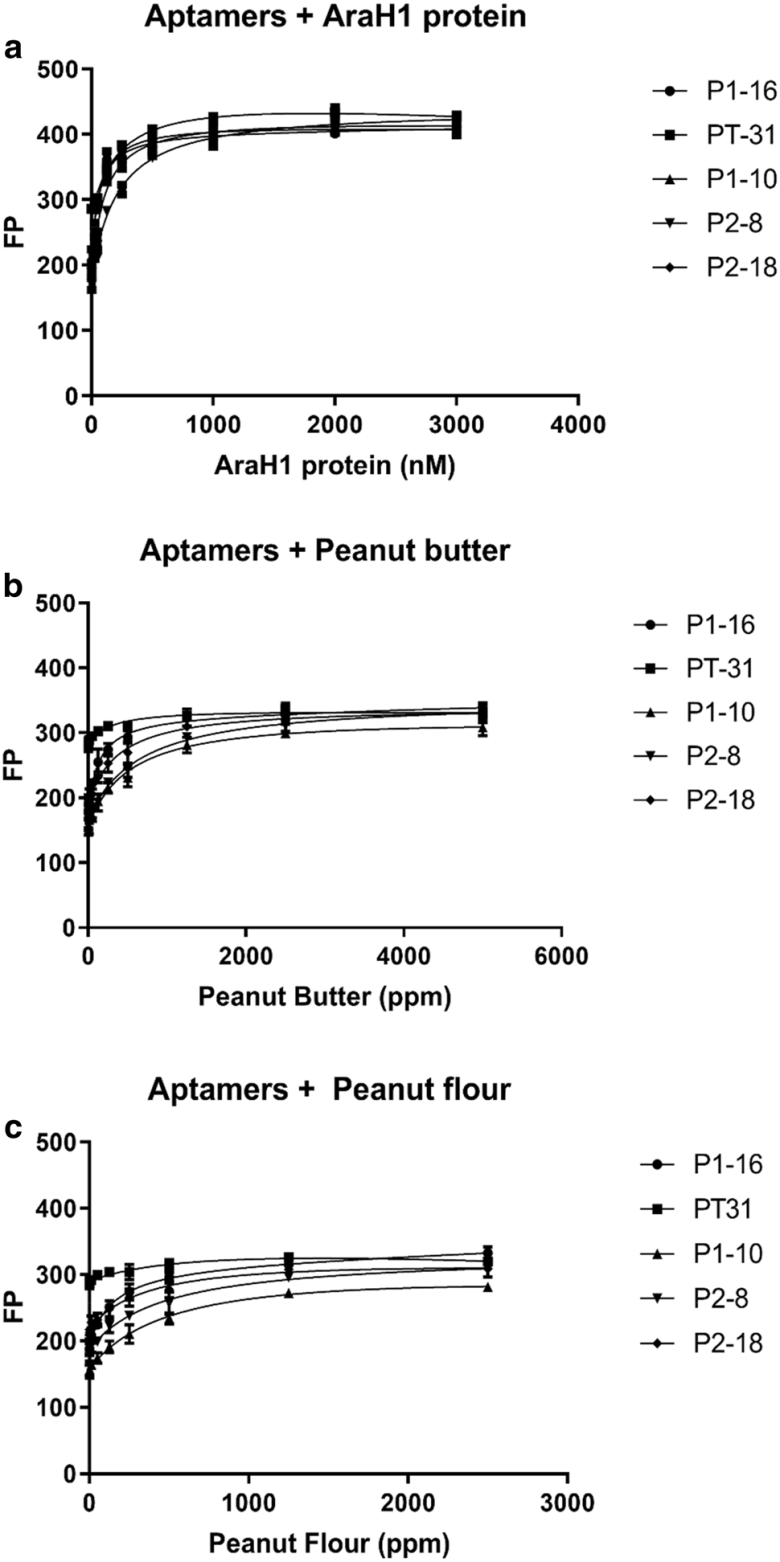
Determination of dissociation constants (K_d_s) for five peanut aptamers and targets. Five aptamers were incubated with increasing concentrations of target purified Ara h 1 protein to determine the K_d_ by fluorescence polarization. (**a**) Purified Ara h 1, (**b**) Peanut butter, (**c**) Peanut flour. Five independent replicates were tested and fitting of the binding isotherm yielded K_d_ values shown in SI Table 1. Error bars represent the standard deviation of the mean.

To determine the affinity of each optimized aptamer for Ara h 1, a major peanut allergen and the most abundant allergen in peanut (12–16% of the total protein content)^26,27^, increasing amounts of purified unlabeled Ara h 1 were incubated with each aptamer and analyzed using fluorescence polarization (FP) to screen for binding affinity (Fig. 1a). The TR-labeled P1-16 (TR-P1-16) aptamer yielded the highest affinity for Ara h 1 (K_d_ ~ 54 ± 5.5 nM) followed by P1-10 and P2-18 (SI Table 1).

To determine whether the aptamers could detect the presence of Ara h 1 in processed peanut, we incubated the aptamers with commercially available peanut flour or peanut butter and repeated the affinity measurements. As observed with purified Ara h 1, TR-P1-16 aptamer yielded a higher affinity for Ara h 1 in peanut butter and peanut flour (K_d_ ~ 141 ± 21.9 ppm, and 144 ± 31.4 ppm, respectively) when compared to the other four aptamers (Fig. 1b,c). Protein, the allergy triggering component, makes up ~ 25% of the peanut commodity and measured by ELISA, the amount of accessible Ara H 1 in the peanut flour was 1.8% by mass (SI Fig. 1).

### Assay design

**Figure 2:**
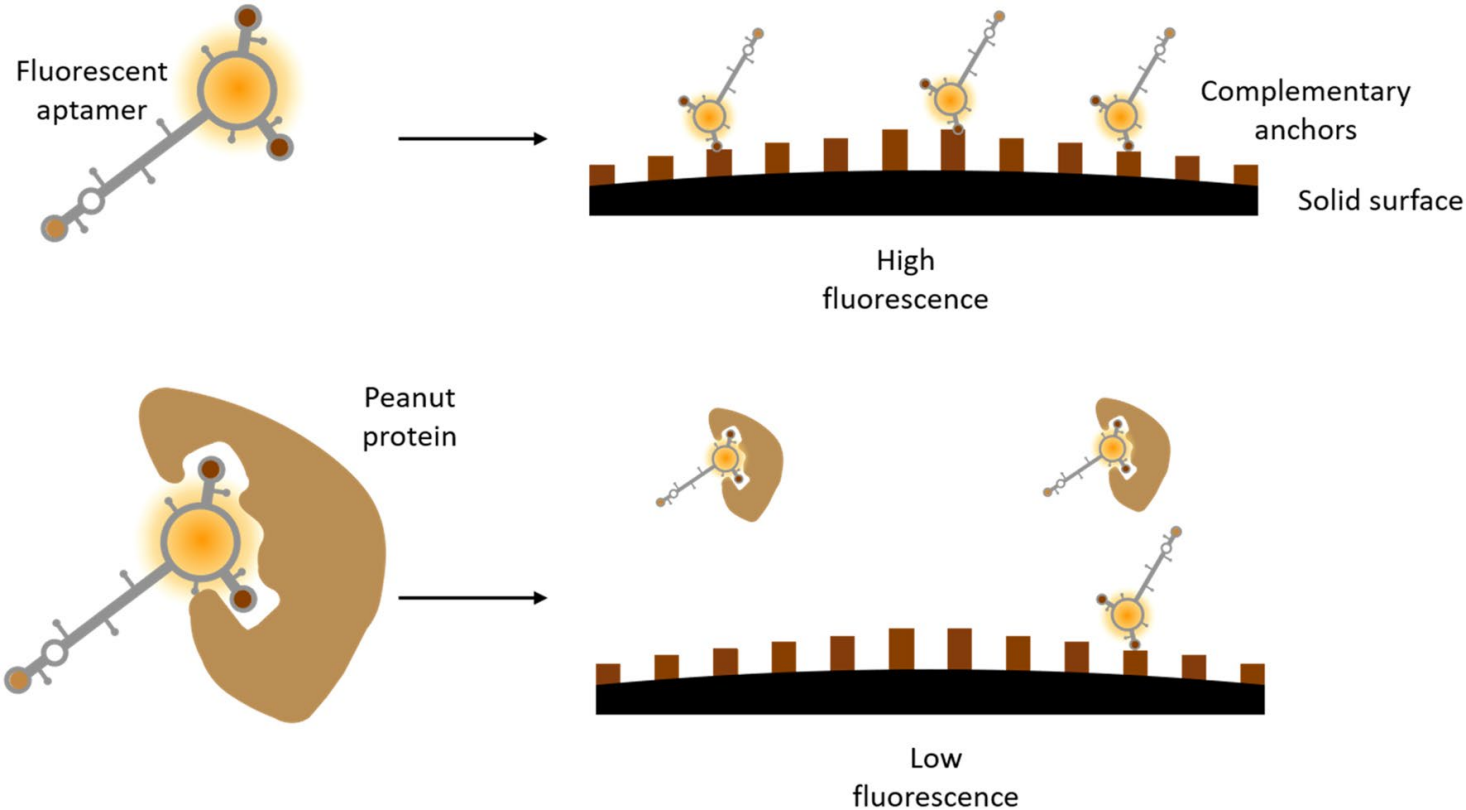
Principles of the assay. Fluorescently labeled aptamer is incubated with sample. If it binds its target, it cannot bind its complementary anchor on the solid surface, leading to low fluorescence.

FP is sensitive to viscosity, temperature, and motion effects, and can be affected by auto-fluorescence of the test matrix^28,29^. To overcome these limitations, we designed a robust assay utilizing short complementary sequences (“anchors”) that are attached to a solid support (Fig. 2). In this assay, the fluorescently labeled aptamer is incubated with the food sample to be tested and, subsequently, applied to the solid support with immobilized anchor sequences. If the aptamer is bound to peanut antigen, it cannot bind to the anchor, and is removed during a subsequent washing step. High fluorescence detected on the support surface therefore signals the absence of peanut antigen (labeled aptamer binds the anchor), and low fluorescence occurs when peanut antigen is present (labeled aptamer is not bound to the anchor).

To select the best anchor sequence for the application, 40 short DNA sequences (anchors), complementary to various regions of the P1-16 aptamer, were covalently attached to an optically clear glass surface. They differed by oligonucleotide sequence, length, or composition/length of the linker adjacent to the surface (carbon atoms or poly-A tail; SI Fig. 2). To facilitate the microarray screening, the aptamers were conjugated to a Cyanine 5 (Cy5) fluorophore rather than Texas Red. After incubation of Cy5-labeled aptamers with homogenized peanut flour, the peanut flour-aptamer mix was added to wells containing the 40 complementary anchors immobilized to glass. After incubation and washing, we detected a decrease in Cy5 fluorescence associated with an anchor complementary to a loop region of the Cy5-P1-16 aptamer. Dilution experiments showed the signal was dependent on the concentration of peanut flour with sensitivity as low as 50 ppm peanut flour (equivalent to 12.5 ppm peanut protein). The poly-A linker was determined to increase the sensitivity as well as the fluorescent baseline (SI Fig. 2). These results were replicated when the anchor was extended with an additional six carbons, suggesting the positioning of the anchor relative to the glass surface influences aptamer binding.

**Figure 3:**
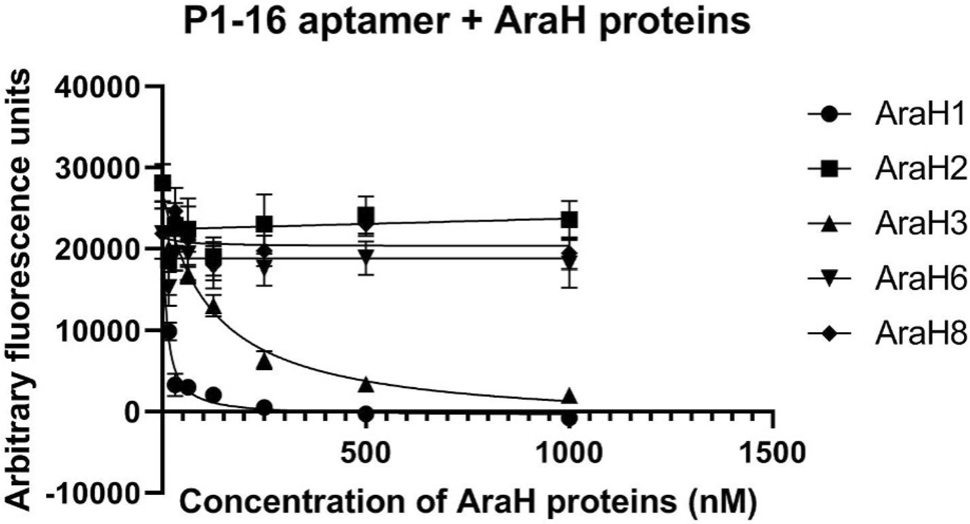
Alexa Fluor 647-labeled P1-16 aptamer binds to Ara h 1 and Ara h 3. Binding specificity was assessed by incubating purified AraH proteins, Ara h 1, Ara h 2, Ara h 3, Ara h 6, and Ara h 8, with AF647-P1-16 aptamer and testing with the benchtop assay. Curve fitting was performed using non-linear regression analysis. Four replicates were tested for each concentration with error bars representing the standard deviation of the mean.

Functionality of the assay using the selected anchor was confirmed by determining specificity of the P1-16 aptamer to various Ara h proteins. While the microarray-based anchor selection was completed with a Cy5 conjugated aptamer, we developed an aptamer modified with the Alexa Fluor 647 (AF647) fluorophore for specificity assays. This modification was made to maximize stability and brightness. Consistent with the FP data, fluorescent intensity associated with AF647-P1-16 decreased with increasing concentrations of Ara h 1 and Ara h 3 but not other peanut proteins (Ara h 2, Ara h 6, and Ara h 8) (Fig. 3). The finding that AF647-P1-16 binds to both Ara h 1 and Ara h 3 is not surprising given that these proteins are members of the cupin superfamily and are structurally similar, with a root mean square deviation (r.m.s.d) of only 2.4 Å when their crystal structures are aligned^30–33^.

To transform the technology into a suitable tool for use by allergic individuals to help manage food consumption choices, we integrated the assay into a small single-use reaction capsule that is run on a durable instrument (SI Fig. 3a). The device was designed to receive small food samples (0.1 g) in a capsule containing the AF647-P1-16 aptamer and homogenization buffer (see “Materials and methods”). The capsule uses a small blender to homogenize samples with buffer, and then passes the homogenate through a polyethylene terephthalate (PET) mesh filter to remove large particulates. The filtered homogenate then flows, via a fluidic sequence, through a reaction chamber (SI Fig. 3b), where the anchor sequences are bound. After rapid incubation (45–90 s, variable by sample), the aptamer–homogenate mixture is washed away, and the reaction chamber is imaged by a camera in the instrument. An image analysis algorithm detects and interprets fluorescence from the remaining bound aptamer and produces the result that indicates whether peanut was or was not detected in the sample. The capsule-based homogenization process was compared to homogenization using lab grade equipment (gentleMACS dissociator set to extract protein mode) and centrifugation (5000*g* for 3 min). When comparing the amount of Ara h 1 extracted from peanut flour using a bench top assay we saw that both methods were similar (70% recovery of Ara h 1 in pod vs gentleMACS, SI Fig. 1).

**Figure 4:**
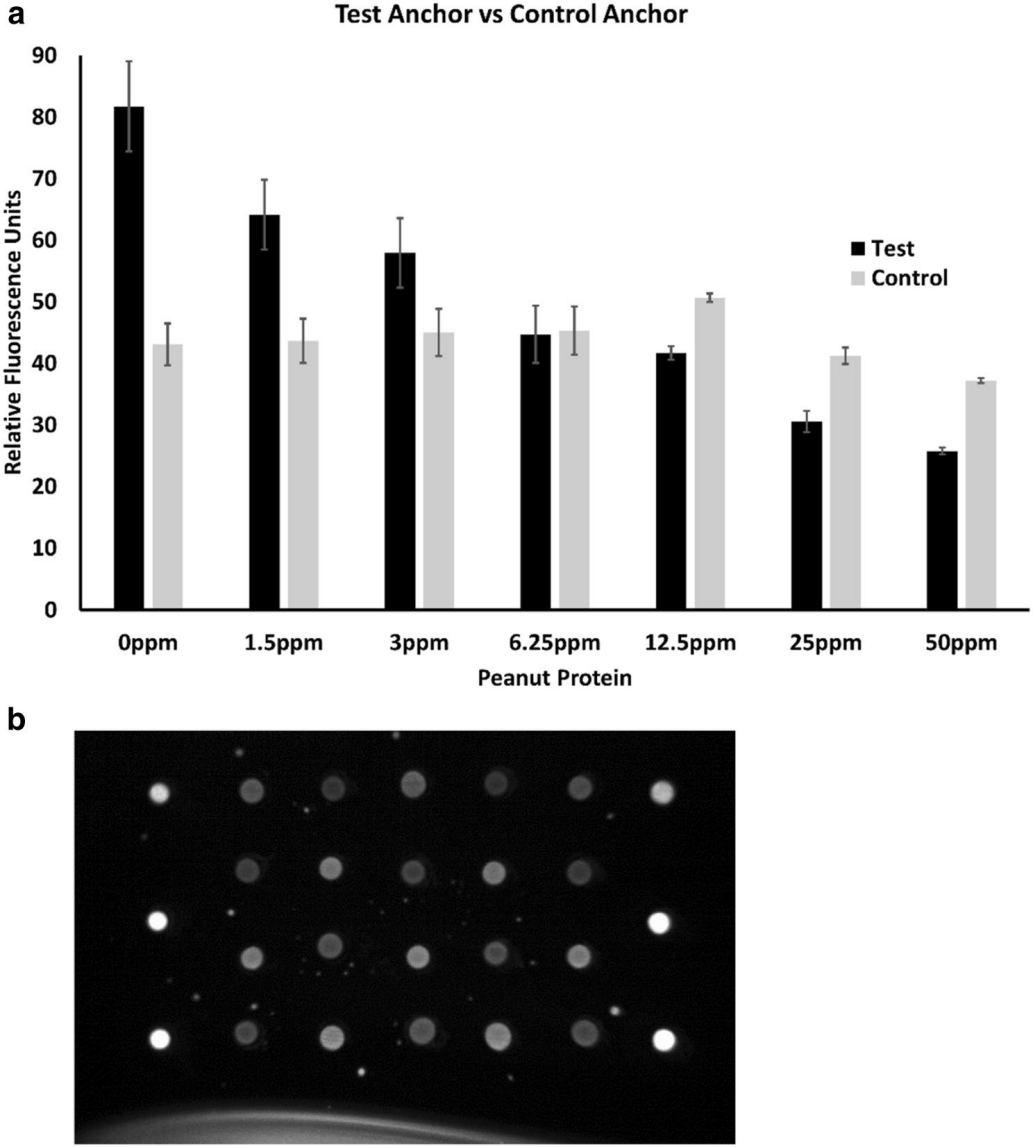
AF647-P1-16 aptamer binds to control anchor with or without peanut in the sample. (**a**) Comparison of AF647-P1-16 aptamer binding to two different anchors (test and control) spotted on the same surface in the presence of increasing concentrations of clarified peanut flour homogenate. Five replicates of each peanut protein concentration were tested. Error bars represent the standard error of the mean. (**b**) Representative image of AF647-P1-16 aptamer bound to both the test spots (top left and alternating) and the control spots. The brighter spots on the left and right sides represent alignment markers for optical performance.

To improve robustness of the assay, we designed an approach to normalize the fluorescent signal to an internal control. We searched our initial collection of 40 anchor sequences (SI Fig. 2) for an anchor complimentary to a second region of the P1-16 aptamer. We confirmed that the selected sequence was not complementary to the test anchor. Control anchors were tested to confirm they were not reactive to peanut by performing AF647-P1-16 aptamer binding tests in the presence of peanut flour homogenate. The top candidate was assayed with the test and control anchors spotted on the same surface in the presence of increasing concentrations of clarified peanut flour homogenate. Five replicates of each peanut protein concentration were tested. Error bars represent the standard error of the mean (Fig. 4a). The bar graph quantifies the intensity of the test spot (black bars) and the control spot (grey bars) in the presence of increasing levels of peanut protein. Comparing control spot intensity across all peanut protein concentrations by one-way ANOVA revealed that there was not a statistically significant difference in control spot intensity in the presence of different peanut concentrations (F-between groups = 2.099, P < 0.9), whereas the test spot did vary according to the presence of peanut protein (F 18.9 P < 1.1 × 10^−9^). These data indicate that the control panel intensity is not influenced by the presence of peanut protein. Spots of the insensitive “control” anchors and peanut-sensitive “test” anchors were arranged in a checkerboard pattern (Fig. 4b) on the solid surface of the reaction capsule. This arrangement was chosen to control for debris or uneven flow and/or uneven illumination of the reaction chamber (SI Fig. 4). Using the control anchor, results of aptamer binding can be reported as (1 – intensity of test/intensity of control) to yield a single value comparable across a variety of food matrices.

### Assay performance

**Figure 5:**
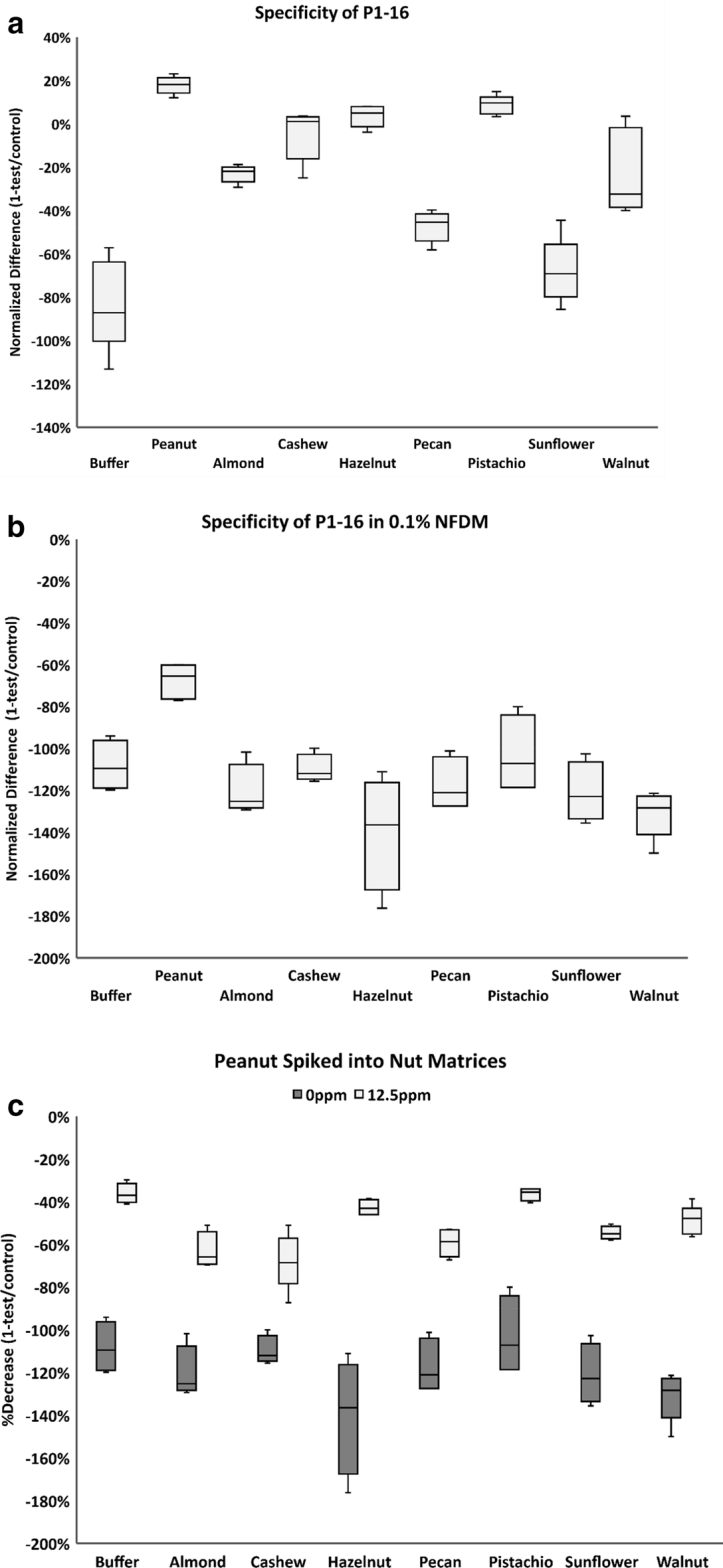
Specificity against tree nuts. (**a**) AF647-P1-16 aptamer binds to peanut protein(s) preferentially to tree nuts, as indicated by the lower normalized difference of tree nut values versus peanut. (**b**) The addition of 0.1% milk added to the buffer as a non-specific food matrix changed the normalized differences of all assays and increased aptamer specificity for peanut versus the other tree nuts. (**c**) AF647-P1-16 aptamer was incubated with clarified tree nut homogenate at 50 ppm nut flour (or control buffer) and spiked with 0 or 12.5 ppm peanut protein. The percent decrease for each run was calculated as 1-(test intensity/control intensity)/100. Four or five replicates were tested for each concentration. Data are presented as a box and whisker plot, with the center line denoting the median value (50th percentile), while the box contains the 25th to 75th percentiles of dataset. The whiskers mark the 5th and 95th percentiles.

To maximize stability and performance of the aptamer in the consumer use format, we moved forward with the AF647 fluorophore modified aptamer. To determine performance and specificity of the assay, we tested the AF647-P1-16 aptamer against multiple types of tree nuts to gauge reactivity towards foods containing proteins of the cupin superfamily^34^. Commercially available almond (*Prunis dulcis*), cashew (*Anacardium occidentale*), hazelnut (*Corylus avellana*), pecan (*Carya illinoinensis*), pistachio (*Pistacia vera*), sunflower (*Helianthus annuus*), and walnut (*Juglans regia*) flours were tested at 0 ppm and 50 ppm and compared to peanut flour. The amount of protein in the nut flours differs by variety, so concentration of the commodity was held constant. As shown in Fig. 5a, the normalized difference between the test and control spots increased relative to buffer in the presence of all samples tested, peanut and the samples containing 50 ppm of pure tree nut flour in buffer. To test whether these data indicate true cross-reactivity or if non-specific interactions with the tree nuts could be eliminated with the addition of a non-specific food matrix, we added 0.1% non-fat dry milk to the assay. In the presence of matrix, the normalized difference between the test and control spots for peanut remained increased compared to buffer, whereas the normalized differences for the tree nut samples decreased relative to buffer (Fig. 5b). These results suggest that in the consumer assay format, where all tests will be performed in the presence of matrix (i.e. ground up food samples), tree nuts are likely to perform similarly to buffer. We also performed a competition experiment by spiking peanut flour with the same concentration of tree nut flours to gauge whether peanut protein could compete with tree nut protein for AF647-P1-16 binding (Fig. 5c). The increase in the normalized differences for the samples containing peanut alone and peanut mixed with tree nut flours (light grey bars) as compared to buffer and samples containing only the nut flours (dark grey bars) clearly show that AF647-P1-16 is responsive to peanut protein in the presence of the tested tree nuts.

**Figure 6:**
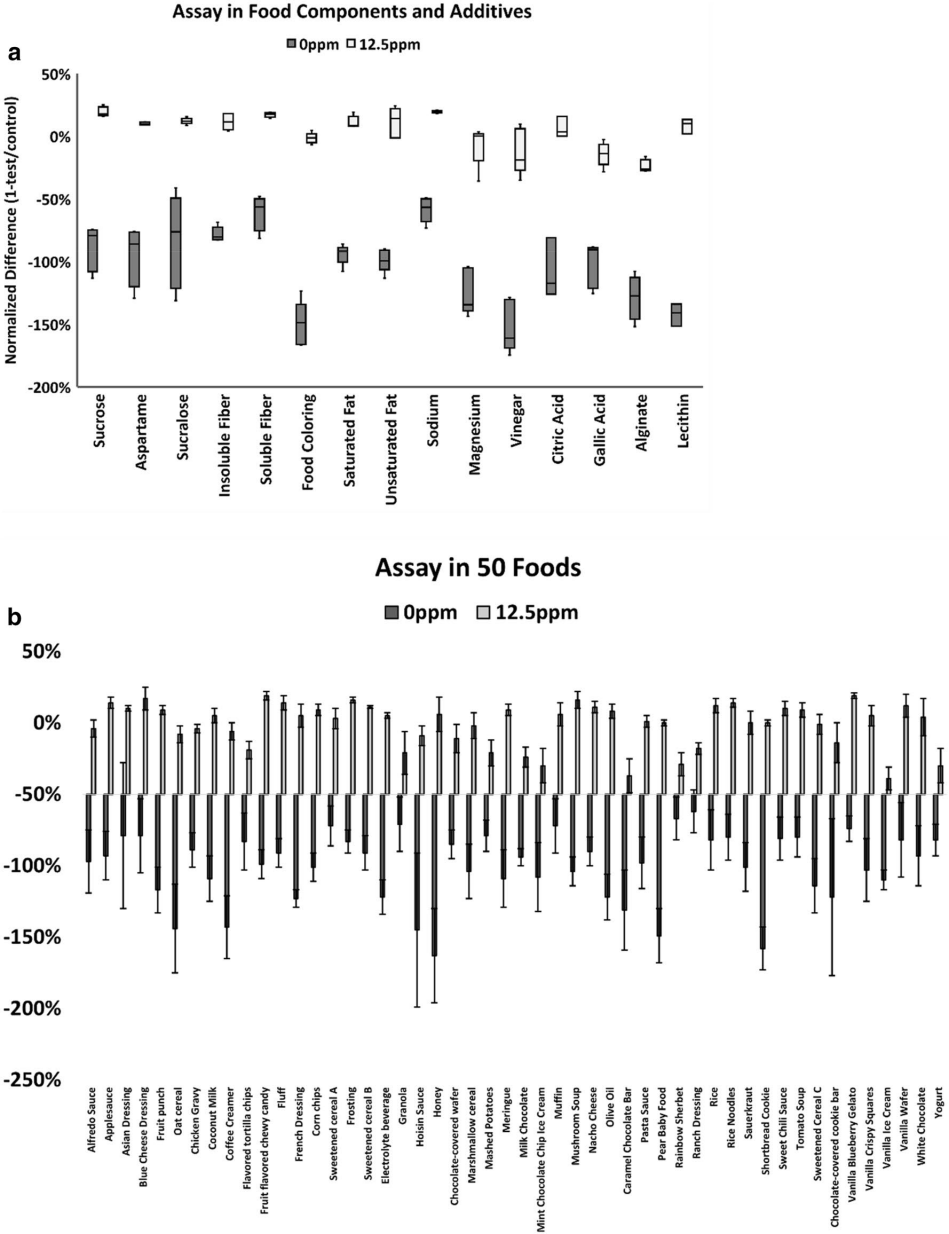
Assay Validation. (**a**) Peanut can be detected in major food components and common food additives. Assay was run using multiple food components and additives, both with and without 12.5 ppm of peanut protein. Four or five replicates of each peanut flour concentration were tested. Data are presented as a box and whisker plot, with the center line denoting the median value (50th percentile), while the box contains the 25th to 75th percentiles of dataset. The whiskers mark the 5th and 95th percentiles. (**b**) Food samples with and without peanut protein can be differentiated by comparing intensity of test spots to control spots. Fifty commercially available foods, spiked with 0 ppm or 12.5 ppm peanut protein, at least 5 replicates each, were tested with AF647-P1-16 aptamer. Using − 50% as an empirically determined cut off value, normalized differences (1 – test/control) are plotted as bars to show clear distinction between food samples containing peanut and those without; the error bars represent standard deviation. All foods containing peanut (at 12.5 ppm) show a normalized difference higher than the defined cut off of − 50% while all the foods that do not contain peanut (0 ppm) show a normalized difference lower than the defined cut off of − 50%.

A robust assay retains sensitivity regardless of the matrix analyzed, therefore, we performed a guard band study on individual ingredients to investigate the effects of potentially high-risk food components and additives (e.g., fats, acid). Tests were conducted at the highest level of these potentially deleterious matrix components typically seen in foods as reported by the USDA in the presence and absence of 12.5 ppm peanut protein (SI Table 2). As shown in Fig. 6a, assay performance was unaffected by most of the components tested, including common sweeteners, insoluble fiber, food coloring, acid, salts, and tannins. Importantly, the assay is robust to high levels of fat; both saturated and unsaturated fat do not negatively impact assay performance (Fig. 6a). Presence of alginate (a common thickening agent) is tolerated in the assay at a concentration as high as 0.01%, a common concentration found in foods. Some foods, such as some ice creams and restructured meats, can have levels exceeding 0.01% alginate. We therefore tested 0.1% alginate and saw decreased sensitivity to peanut protein (data not shown). While high concentration of alginate in buffer impacted our assay, the results may not always predict poor performance in complex food matrices. For example, ice cream containing alginate performs well in our assay (Fig. 6b).

To confirm the accuracy of our integrated device, we tested 50 foods that represent 14 food categories suggested by AOAC International for quantitation of peanut by ELISA-based methods as well as foods identified by peanut allergic individuals as high risk for peanut contamination (Fig. 6b and SI Table 3). For each food, at least four replicates were run without peanut (0 ppm) and at least four replicates were spiked with peanut protein (12.5 ppm, equivalent to 37.5 µg of peanut protein in a 100 mg food sample). Additionally, we tested 20 commercially available foods that were known to contain peanut (SI Table 4). For each food, the normalized difference (1 – test/control) was plotted (Fig. 6b). After setting a threshold value for samples that contain peanut and those that do not, the ability of the assay to detect peanut was confirmed, with an accuracy of 99% (Table 1).

**Table 1:** Results from 495 runs across 50 foods.

	Number of runs	% of runs
True positive	232	99
False negative	2	1
True negative	239	98
False positive	5	2
Sensitivity	99%	
Specificity	98%	
Accuracy	99%	
PPV (positive predictive value)	98%	
NPV (negative predictive value)	99%	
Likelihood ratio of a negative test	48.4	
Likelihood ratio of a positive test	0.01	

### Future work

**Figure 7:**
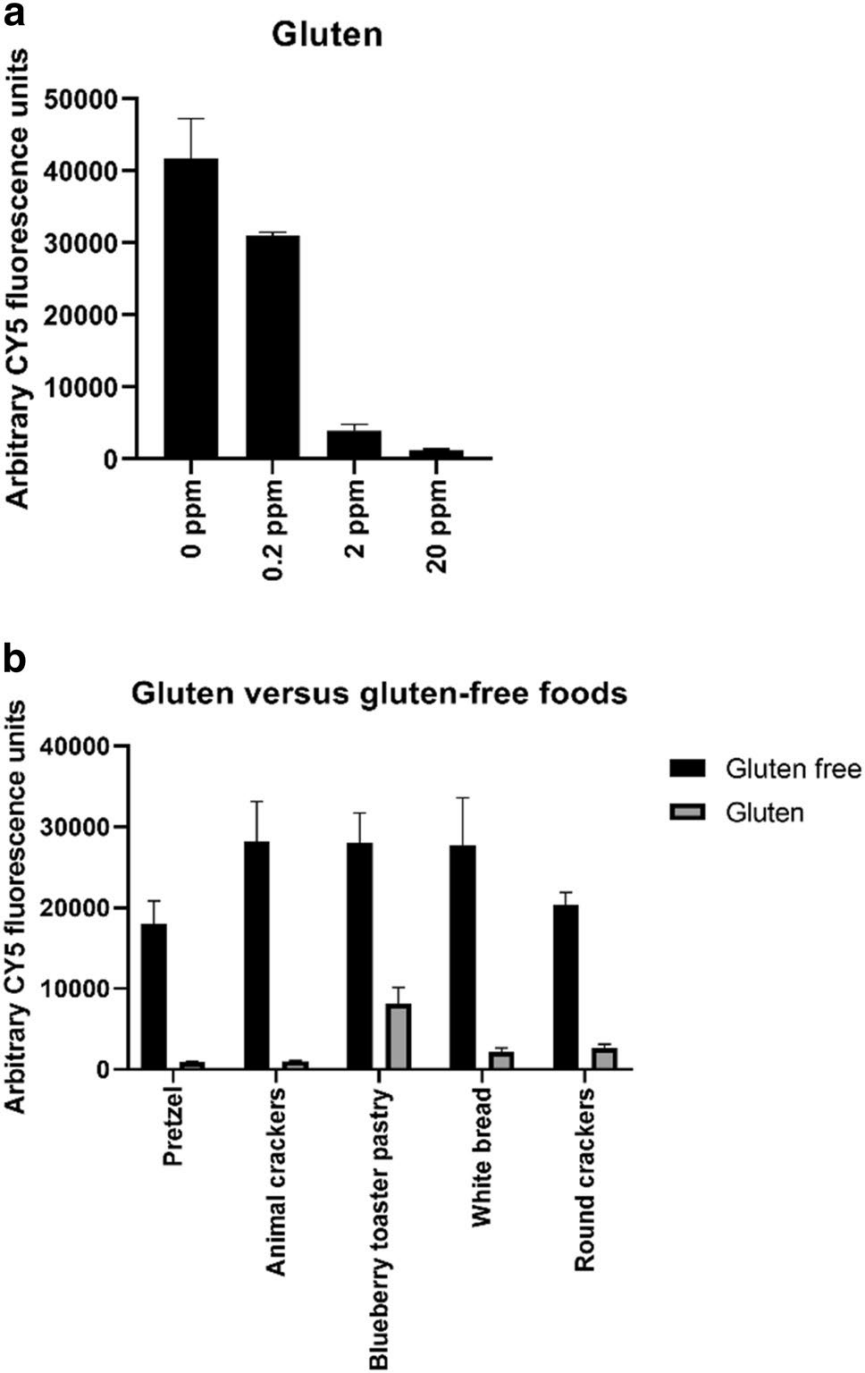
Future work. (**a**) Cy5-GN5 aptamer binds to gluten in a concentration dependent manner and in a variety of food matrices. GN5 aptamer was incubated in buffer spiked with increasing concentrations of gluten. (**b**) Commercially available foods (gluten versus gluten-free) were homogenized, filtered, then incubated with Cy5-GN5. As described for the P1-16 aptamer, the samples were incubated with a chip spotted with a 10 oligonucleotide anchor complementary to sequence of GN5. Four replicates of each sample were tested. Error bars represent the standard deviation of the mean.

This assay can be extended to any target that can be recognized by an aptamer. To demonstrate this, we performed preliminary work on an additional target, gluten. Briefly, aptamers that target gluten were chosen by SELEX and screened to hybridize to anchor sequences as described for the P1-16 aptamer. The selected aptamer (GN5) exhibited high sensitivity to gluten, as shown with a dose-dependent curve (Fig. 7a) showing the fluorescence intensity (Cy5) significantly decreasing in the presence of 0.2 ppm gluten. We also challenged the Cy5-GN5 aptamer against commercially available foods and were able to detect the presence of gluten in commonly consumed foods (Fig. 7b).

## Discussion

Here we describe the design and development of an aptamer-based assay platform with the potential to detect allergens and other targets in a broad spectrum of foods. For detection of peanut antigen, an aptamer, P1-16, was identified and binding affinity determined for pure peanut protein Ara h 1 (K_d_ ~ 54 ± 5.5 nM) and Ara h 1 in peanut butter and peanut flour (K_d_ ~ 141 ± 21.9 ppm, and 144 ± 31.4 ppm, respectively; Fig. 1). We effectively immobilized P1-16 via anchors to optically clear glass (Fig. 2) within a single-use reaction capsule that is run on a consumer-friendly instrument (SI Fig. 3a). Specificity of the assay was determined (Figs. 3, 5) and the reagents tested for stability (SI Fig. 5). Finally, the assembled peanut allergen test successfully detected peanut antigen in 50 foods spanning 14 food categories (Table 1).

In a recent Joint Expert Consultation meeting by FAO/WHO on Risk Assessment of Food Allergens, an Expert Committee established threshold levels in foods of priority allergens below which most allergic consumers would not suffer an adverse reaction. Based on data reported by Remington et al.^35^ and Houben et al.^36^, the Committee recommended a threshold of 2 mg for peanut protein. Our peanut allergen test builds off traditional SELEX approaches^1,25^ to improve assay sensitivity, selectivity, and flexibility^37^ and allow detection of 12.5 ppm peanut protein in foods (equivalent to 37.5 µg within a 100 mg food sample tested in the pod), despite the challenges of antigen detection in complex matrices, e.g., high protein, high salt, or acidity. Key to the approach is use of two different anchor sequences, (1) the test anchor, which competes with peanut protein for binding to P1-16 and (2) a control anchor that binds a different region of the P1-16 aptamer and is not competitive with peanut protein binding. The control anchor thus serves as a matrix condition control (Fig. 4A). Updating the assay to image the fluorescent intensities of the test and control spots separately allowed the signal output to be normalized (1 – intensity of test/intensity of control) to yield a single value that could be compared across a variety of food matrices.

Our aptamer assay features several practical advantages, including reagents that are (1) synthesized and chemically modified in a fast, reproducible, and scalable process; (2) small and inexpensive to manufacture with reproducible production characteristics; and (3) stable over a range of temperatures and pH values. The all-in-one detection platform also represents an end-to-end solution, involving (1) sample collection, (2) homogenization and filtration methods, including a universal extraction step that can be applied to food as well as other matrices (serum, saliva, etc.), and (3) a precision optical sensor and algorithm with built-in controls. As an added advantage, we demonstrate that our aptamer approach can be readily modified to detect additional targets, such as gluten, and is suited to rapid transition to additional food allergens as well as other targets such as pesticides and even heavy metals.

Previous research has tried to utilize aptamer-based sensor for the detection of peanut protein^7–10^. All sensors produced to date have reported detection of purified Ara h 1 at concentrations ranging from 0.02 to 28 ppm, with process steps that take more than 30 min to complete. In this paper we show for the first time an aptamer-based protein detection system that can extract and detect peanut protein in under 3 min, also we have proven the ability to detect 12.5 ppm peanut protein (1.8 ppm Ara h 1) in 50 spiked food matrices and 20 foods containing peanut as an ingredient. These data together show, for the first time, a sensor that has the performance necessary for a consumer application and demonstrate the function of our all-in-one detection platform for end-to-end sample processing and detection.

## Materials and methods

### Aptamer selection

Aptamer selection was performed using GO-SELEX^38^. Briefly, the SELEX selection process begins with a random library of single-stranded DNA (ssDNA). Each 76 base pair ssDNA is comprised of a central 30 base pair sequence, which is randomly distributed, with two ends (23 bp each) of known sequences to serve as primers (SI Table 8). In the first round of SELEX, the ssDNA from the random pool is diluted to 20 ng/µl in water. Peanut protein (or gluten) is mixed in extraction/assay buffer (20 mM EPPS, 0.2% Brij-58, 2% PEG 8000, 2% Pluronic F-127, pH 8.4), 15 nM AF647-P1-16, and 30 mM MgCl_2_) and diluted to the desired concentration, which is dependent on the round. 100 µl of diluted ssDNA is added to 300 µl of diluted peanut protein, and the resulting mixture incubated at room temperature with shaking for a set amount of time dictated by the round (SI Tables 5, 6). This ssDNA/protein mixture is added to 600µL of graphene oxide (GO), incubated for 20 min at room temperature with shaking to remove ssDNA that is not bound to protein, and centrifuged at 10,000*g* for 3 min. The supernatant, containing ssDNA bound to the target protein, is cleaned by adding 10% Strataclean resin and heating to 80 °C for 3 min, followed by centrifugation at 10,000*g* for 3 min. The supernatant is collected, and the Strataclean step repeated. The concentration of ssDNA in the final supernatant is measured and compared to the initial concentration prior to addition of protein and GO, to define percent recovery. The cleaned ssDNA pool is then amplified by PCR using a biotinylated reverse primer using standard cycling procedures and the product cleaned using the ChargeSwitch Pro PCR Clean Up Kit. To regenerate ssDNA for the next round, clean PCR product is added to streptavidin coated magnetic beads (Dynabeads MyOne Streptavidin C1) and base is added to denature the dsDNA. Resulting ssDNA is collected using the magnetic beads and concentrated using an Amicon Ultra 3 k Spin Column. Subsequent round of SELEX are performed on the resulting ssDNA. SELEX rounds performed for peanut protein and gluten are presented in SI Tables 5 and 6, respectively. After SELEX selection, ssDNAs were cleaned using a ChargeSwitch Pro PCR Clean Up Kit (Thermo Fisher Scientific) and sent to deep sequencing (Illumina Miseq, UMass Deep Sequencing Core). A heatmap of the sequences was created using Galaxy (open-source bioinformatics tools), which indicated that the top 40 sequences could be divided into 5 families based on a consistent core sequence (SI Table 7). The top sequence from each of the families was synthesized and tested for binding affinity to peanut protein as shown in Fig. 1.

### Aptamer modifications

To prepare aptamers for incorporation into a chip-based assay we added either C-Rich or G-rich supplemental sequences to the 5′ and 3′ ends. The sequences chosen were based on the 5’ and 3’ ends of the selection library and modified to be G/C rich to improve binding to the anchors. These sequences were folded (using IDT oligo-analyzer and M-fold) and analyzed for similarities and significant 3-dimensional structure. From those data, 10–15 sequences were synthesized and screened for Kd based on FP (Fig. 1—data shown for top 5 sequences representing the 5 families). 5′ and 3′ sequences of the selected aptamers are shown in SI Table 8.

Aptamers were modified with fluorophores chosen for compatibility with assays performed throughout the study. All three fluorophores have similar excitation and emission spectrums; Texas Red (625 g/mol, Em.640 nm, Ex. 580 nm), Alexa647 (1155 g/mol, Em.665, Ex 650), and Cy5 (792 g/mol, em. 670 nm Ex. 651 nm). Texas Red was used in FP assays, since it is the smallest molecule with the least interference to Brownian motion, with a shorter relaxation time, and is commonly used in FP studies^39^. To transition the assay to chip format, we moved towards CY5 which is commonly used in microarray assays. As our development progressed and we aimed to create a consumer product that is stable at room temperature for long periods of time we decided to shift toward Alexa647^40^, which is more resistant to photobleaching over time.

### Affinity measurements

Fluorescence polarization (FP) was used to determine dissociation constants (K_d_s) for PT-31, P1-10, P1-16, P2-8, and P2-18 interaction with potential targets. The aptamers were synthesized from Integrated DNA Technologies with a Texas Red fluorophore attached to its 5’ end in order to measure changes in fluorescence polarization. Each experiment was performed on a TECAN Spark 10 M plate reader (excitation 570 nm/emission 625 nm) set to 5 kinetic cycles. Samples were prepared in 50 µL with FP buffer (50 mM Tris–HCl, 0.1% Tween-20, pH 9) containing 5 nM aptamer and increasing concentration of purified AraH1 (Indoor Biotechnologies) or peanut matrix (Teddie brand unsalted peanut butter or Protein Plus brand roasted natural peanut flour) ranging from 0 to 50 µM and incubated for 10 min prior to reading on the spectrofluorometer. Peanut matrices were prepared by homogenizing samples at a stock concentration of 100,000 parts per million in FP buffer and clarifying by centrifugation at 5000×*g* for three minutes. Nonlinear regression analyses were used to determine K_d_s (Prism 8, GraphPad).

### Anchor screening

CY5-labeled aptamers and 40 short DNA anchors with a ten-oligonucleotide sequence complementary to the aptamers were synthesized at Integrated DNA Technologies. Half of the anchor sequences contained a poly-A tail and all anchor sequences contained an amine linker at the 5’ end of the oligonucleotide. Each anchor was spotted on epoxy silane-treated slides at various concentrations (1–40 µM) at Applied Microarrays (Tempe, Arizona). Each slide was pre-blocked with 1% bovine serum albumin in HEPES for two minutes prior to incubation of CY5-aptamer/peanut flour mixture. Aptamer was mixed with peanut flour at different concentrations and allowed to incubate in binding buffer prior to loading onto the well. After a two-minute incubation with mild shaking on an orbital rotator, the slides were washed with binding buffer and scanned for fluorescent CY5 signal. After selection of the 5′-(Amine-6C)-anchor, the linker was extended by six additional carbon atoms and printed at a concentration of 5 µM on epoxy silane-treated slides for confirmation.

### Assay details

COP chips were placed in an air-tight chamber with fluidic channels connected to reservoirs from which wash solution or filtered food homogenate were drawn via a pump system. First, 100 µL of wash solution (20 mM Trizma base, 0.2% Brij-L4, 0.2% Capstone FS-31, 0.25 mM MgCl_2_) was delivered to the chamber, followed by a short air purge. Then, 100 µL of test sample (containing homogenization buffer (20 mM EPPS, 0.2% Brij-58, 2% PEG 8000, 2% Pluronic F-127, pH 8.4), 15 nM AF647-P1-16, and 30 mM MgCl_2_) was delivered to the chamber at a rate of 1000 µL/min. The chamber was cleared, an image captured, and the intensity of the control spots assessed. If less than 30 rfu, another aliquot of 100 µL of test sample was delivered. The process was repeated until the intensity of the control spots is greater than 30 rfu. Then the chamber was washed with 200 µL of wash solution and imaged.

### Guard band study

For the matrix interference studies, 15 nM AF647-P1-16 was incubated briefly with the listed additives and components in homogenization buffer (20 mM EPPS, 2% Pluronic F-127, 2% PEG 8000, 0.2% Brij-58, pH 8.4). The percentage represents the amount in a food sample, meaning for a value of 100%, 0.1 g of component was added to 3 mL of assay buffer. Peanut flour was then added to the 50 ppm samples, and the assay was run as described above.

### Specificity studies

AF647-P1-16 aptamer (20 nM) was incubated with increasing concentrations of purified AraH proteins (Indoor Biotechnologies, AraH1, #NA-AH1-1; AraH2, #NA-AH2-1; AraH3, #NA-AH3-1; AraH6, #NA-AH6-1; AraH8, #RP-AH8-1) in assay buffer and 30 mM MgCl_2_. Commercially available nut flours (pecan, walnut, pistachio, hazelnut, almond, sunflower, and cashew) were homogenized with assay buffer and clarified by centrifugation at 5000×*g* for three minutes. To study the specificity of P1-16 aptamer when tree nut is present, 0 or 50 ppm peanut flour was spiked into clarified 50 ppm tree nut flour in assay buffer or 0.1% non-fat milk (dry powder, American Bio). Samples were assayed as described for the guard band study.

### Matrix testing to validate assay

Chips were printed with both 12.5 µM P1-16 anchor and 7 µM of the control anchor. Foods were sampled at 0.1 g and homogenized for 45 s in 3 mL of assay buffer with 7.5 nM P1-16. For peanut containing samples, 30 µL of 5000 ppm peanut homogenate was also added. Finally, 23 mM MgCl_2_ was added to all samples. The assay ran as described above. The foods tested were: vanilla ice cream, sugar-free vanilla wafer, gelato, milk chocolate, mint chocolate chip ice cream, nacho cheese, pasta sauce, mushroom soup, sweetened cereal, white chocolate, applesauce, oat cereal, chicken gravy, hoisin sauce, packaged cupcakes, rice noodles, vanilla crispy squares, blue cheese dressing, alfredo sauce, frosting, pink meringue cookie, fluff, marshmallow cereal, sauerkraut, fruit flavored chewy candy, Asian dressing, fruit punch, coconut milk, coffee creamer, flavored tortilla chips, French dressing, corn chips, sweetened cereal, electrolyte beverage, granola, honey, chocolate-covered wafer, mashed potatoes, olive oil, pear baby food, rainbow sherbet, ranch dressing, white rice, shortbread cookie, sweet red chili sauce, tomato soup, chocolate-covered cookie bar, and yogurt.

### Gluten assay

GN5 aptamer was incubated in gluten assay buffer (GAB, 15.4 mM MES buffer, 0.08% Tween-20, 30% ethanol, and 1 mM MgCl_2_, pH 5) with increasing concentrations of gluten. Gluten (wheat source, Sigma Life Science) was extracted in GAB and diluted in GAB with 20 nM GN5. For the food testing, commercially available foods were paired with the closest match for the gluten-free counterpart: wheat round crackers versus gluten-free round crackers (corn starch and rice flour); wheat frosted blueberry toaster pastry versus gluten-free frosted blueberry toaster pastry (rice flour); wheat pretzel sticks versus gluten free pretzel stick (corn and potato starches); country white bread versus gluten-free white bread (pea, tapioca, and rice starches); animal crackers (wheat) versus gluten-free animal crackers (pea and potato starches) were tested with GN5. Each food was prepared as described in the matrix testing description; after filtration the food filtrate was diluted by an additional 1:10 with GAB. For both assays, 250 µL of GAB is delivered to the chamber, followed by a short air purge. Then, 500 µL of test sample (GAB and 20 nM GN5, with or without gluten) is delivered to the chamber at a rate of 1000 µL/min. Then the chamber was washed with 525 µL of GAB with 10 mM MgCl_2_ with the same flow rate. The chips were then air dried and imaged.

### Long-term stability experiments

AF647-P1-16 (10 nM) was formulated in autoclaved homogenization buffer (20 mM EPPS, 0.2% Brij-58, 2% PEG-8000, 2% Pluronic F-127, pH 8.4) under aseptic, sterile environmental conditions within a clean room facility. Aliquots of such samples were subjected to accelerated aging at 37 °C. At each time point, samples were compared with respect to age-matched fresh P1-16.

## Supplementary Information

Supplementary Information.
