# Supplementary material for: Aptamer based point of care diagnostic for the detection of food allergens
Source: Sci Rep. 2022 Jan 25;12:1303. doi: 10.1038/s41598-022-05265-0 (PMC8789827; doi:10.1038/s41598-022-05265-0)
Supplement: Supplementary file 1 — Supplementary Information. [file 41598_2022_5265_MOESM1_ESM.docx]

**Supporting Information**

|  | **P1-16** | **PT-31** | **P1-10** | **P2-8** | **P2-18** |
| --- | --- | --- | --- | --- | --- |
| AraH1 protein (nM) | 54.4 ± 5.5 | 238.1 ± 35.3 | 69.2 ± 9.4 | 265.8 ± 25.8 | 104.7 ± 13.5 |
| Peanut butter (ppm) | 141 ± 21.9 | 311.3 ± 108.5 | 544.7 ± 110.3 | 650.8 ± 152.7 | 386.5 ± 89.1 |
| Peanut flour (ppm) | 144.3 ± 31.4 | 791.7 ± 648.6 | 557.2 ± 166.6 | 466.1 ± 187.1 | 383.1 ± 143.5 |

SI Table 1. Summary of determination of dissociation constants (K_d_s). K_d_ values shown are mean +/- standard error.

| **Sample** | **OD 450** | **Calculated ng/mL Ara h 1** | **Calculated ppm Ara h 1** |
| --- | --- | --- | --- |
| Peanut flour (100 ppm) – Gentle Macs dissociation | 3.22 (+/- 0.07) | 1820 | 1.8 |
| Peanut flour (100 ppm) – Pod dissociation | 2.26 (+/- 0.05) | 1256 | 1.2 |

| **Analyte** | **Corresponding Concentration (Literature Value)** | **Percentage of Peanut Flour** |
| --- | --- | --- |
| Peanut flour | 50 ppm | --- |
| Peanut protein | 12.5 ppm | 25% |
| Ara h 1 | 1.8 ppm (26 nM) | 3.5% (14% of peanut protein) |

SI Figure 1. Determination of Ara h 1 content in commercially-available peanut flour by ELISA. Analysis performed as directed by Indoor Biotechnologies Ara h 1 ELISA 2.0 kit. Comparison to expected values from the literature.

**A**

**B C**


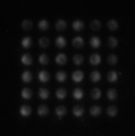


| **Name** | **Anchor Sequence** | **Modification** |
| --- | --- | --- |
| 3_1 | /5AmMC6/aaaaaAATGGTCATC | 5' 6CAmine |
| 3_2 | /5AmMC6/aaaaaAATAAGCGTG | 5' 6CAmine |
| 3_3 | /5AmMC6/aaaaaAAATTCGCAG | 5' 6CAmine |
| 3_4 | /5AmMC6/aaaaaAAATTCGCAC | 5' 6CAmine |
| 5_1 | /5AmMC6/aaaaaACGCCTTCTG | 5' 6CAmine |
| 5_2 | /5AmMC6/aaaaaAGCGTGTAAG | 5' 6CAmine |
| 5_3 | /5AmMC6/aaaaaATCCCTTCTC | 5' 6CAmine |
| 5_4 | /5AmMC6/aaaaaATGGTGTAAG | 5' 6CAmine |
| P1_10 | /5AmMC6/aaaaaTGAGGAGTT | 5' 6CAmine |
| P2_8 | /5AmMC6/aaaaaATCCTCTAA | 5' 6CAmine |
| P2_18 | /5AmMC6/aaaaaATGGGTTGT | 5' 6CAmine |
| AP_1 | /5AmMC6/TTCGCACACA | 5' 6CAmine |
| AP_2 | /5AmMC6/ACACACGGAC | 5' 6CAmine |
| AP_3 | /5AmMC6/CGGACTTACG | 5' 6CAmine |
| AP_4 | /5AmMC6/TTACGGCCCA | 5' 6CAmine |
| AP_5 | /5AmMC6/GCCCACCCAC | 5' 6CAmine |
| AP_6 | /5AmMC6/CCCACAGATT | 5' 6CAmine |
| AP_7 | /5AmMC6/AGATT CGCAT | 5' 6CAmine |
| AP_8 | /5AmMC6/CGCATCCACT | 5' 6CAmine |
| AP_9 | /5AmMC6/CCACTCAGCT | 5' 6CAmine |
| AP_10 | /5AmMC6/CAGCTCGACC | 5' 6CAmine |
| AP_11 | /5AmMC6/CGACCCCCCC | 5' 6CAmine |
| AP_12 | /5AmMC6/CCCCCGGTAG | 5' 6CAmine |
| AP_13 | /5AmMC6/GGTAGAAGCG | 5' 6CAmine |
| AP_14 | /5AmMC6/AAGCGGAATG | 5' 6CAmine |
| AP_15 | /5AmMC6/GAATGTGCGA | 5' 6CAmine |
| AP_1_PA | /5AmMC6/aaaaaTTCGCACACA | 5' 6CAmine |
| AP_2_PA | /5AmMC6/aaaaaACACACGGAC | 5' 6CAmine |
| AP_3_PA | /5AmMC6/aaaaaCGGACTTACG | 5' 6CAmine |
| AP_4_PA | /5AmMC6/aaaaaTTACGGCCCA | 5' 6CAmine |
| AP_5_PA | /5AmMC6/aaaaaGCCCACCCAC | 5' 6CAmine |
| AP_6_PA | /5AmMC6/aaaaaCCCACAGATT | 5' 6CAmine |
| AP_7_PA | /5AmMC6/aaaaaAGATT CGCAT | 5' 6CAmine |
| AP_8_PA | /5AmMC6/aaaaaCGCATCCACT | 5' 6CAmine |
| AP_9_PA | /5AmMC6/aaaaaCCACTCAGCT | 5' 6CAmine |
| AP_10_PA | /5AmMC6/aaaaaCAGCTCGACC | 5' 6CAmine |
| AP_11_PA | /5AmMC6/aaaaaCGACCCCCCC | 5' 6CAmine |
| AP_12_PA | /5AmMC6/aaaaaCCCCCGGTAG | 5' 6CAmine |
| AP_13_PA | /5AmMC6/aaaaaGGTAGAAGCG | 5' 6CAmine |
| AP_14_PA | /5AmMC6/aaaaaAAGCGGAATG | 5' 6CAmine |
| AP_15_PA | /5AmMC6/aaaaaGAATGTGCGA | 5' 6CAmine |

SI Figure 2. AP-PA-1 anchor is the optimal oligonucleotide sequence identified in a microarray screen. A. Forty-one sequences were covalently attached to epoxysilane coated glass slides and screened for their ability to hybridize with CY5-P1-16 aptamer. B. Representative image of CY5-P1-16 aptamer bound to the complement anchor. Each spot was printed with 10 uM anchor. C. From the screen, one anchor was chosen and printed with a longer carbon linker. For A and B, sensitivity was also assessed by incubating CY5-P1-16 aptamer with increasing concentrations of clarified peanut flour homogenate prior to exposure with each anchor. CY5 fluorescence was quantified after washing and drying the slides. Three independent replicates of each concentration were tested with error bars representing the standard deviation of the mean. The anchor sequences tested are provided in the table.

**A B**


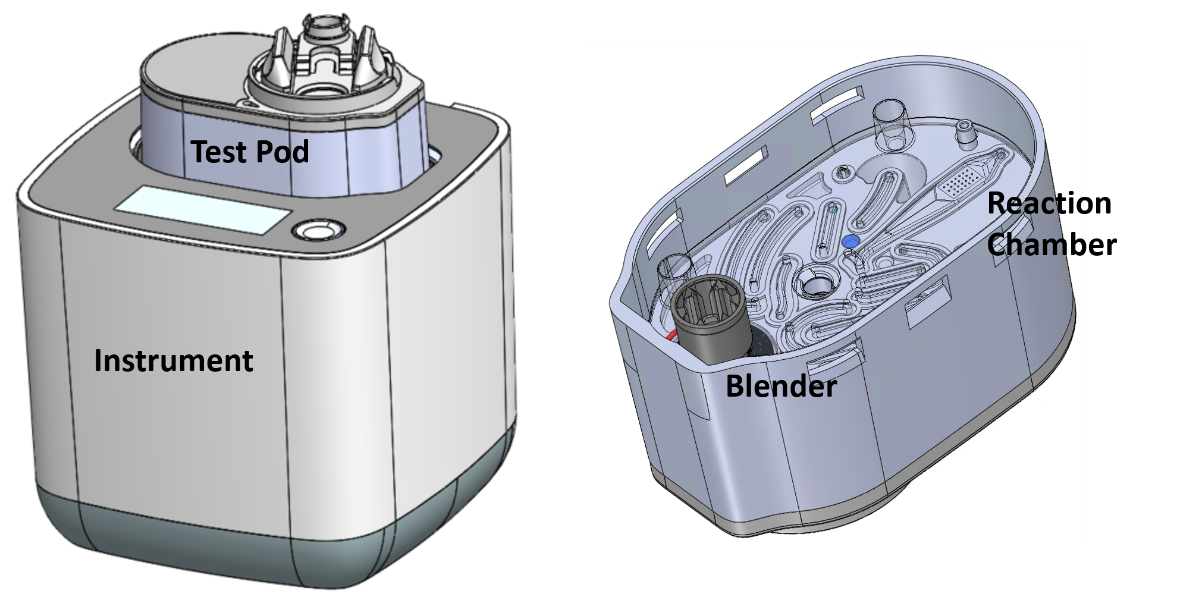


SI Figure 3. Illustrations of the integrated assay test pod and instrument. A. Single-use test pod is driven by the durable instrument. B. Cutaway view of pod shows area where food sample is homogenized and the reaction chamber containing the surface bound anchor sequences.


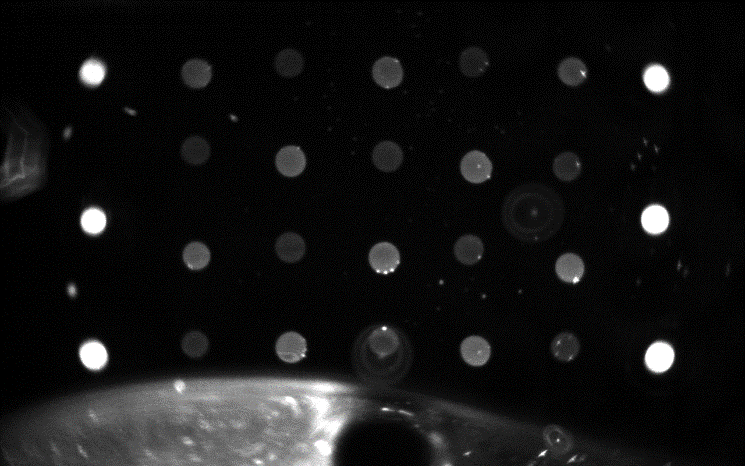


SI Figure 4 – An example of a poor image in which several spots cannot be used due to poor reaction flow (remaining fluid in bottom of image) and particulates of food debris (bright speckles). Poor spots are not considered in final analysis.


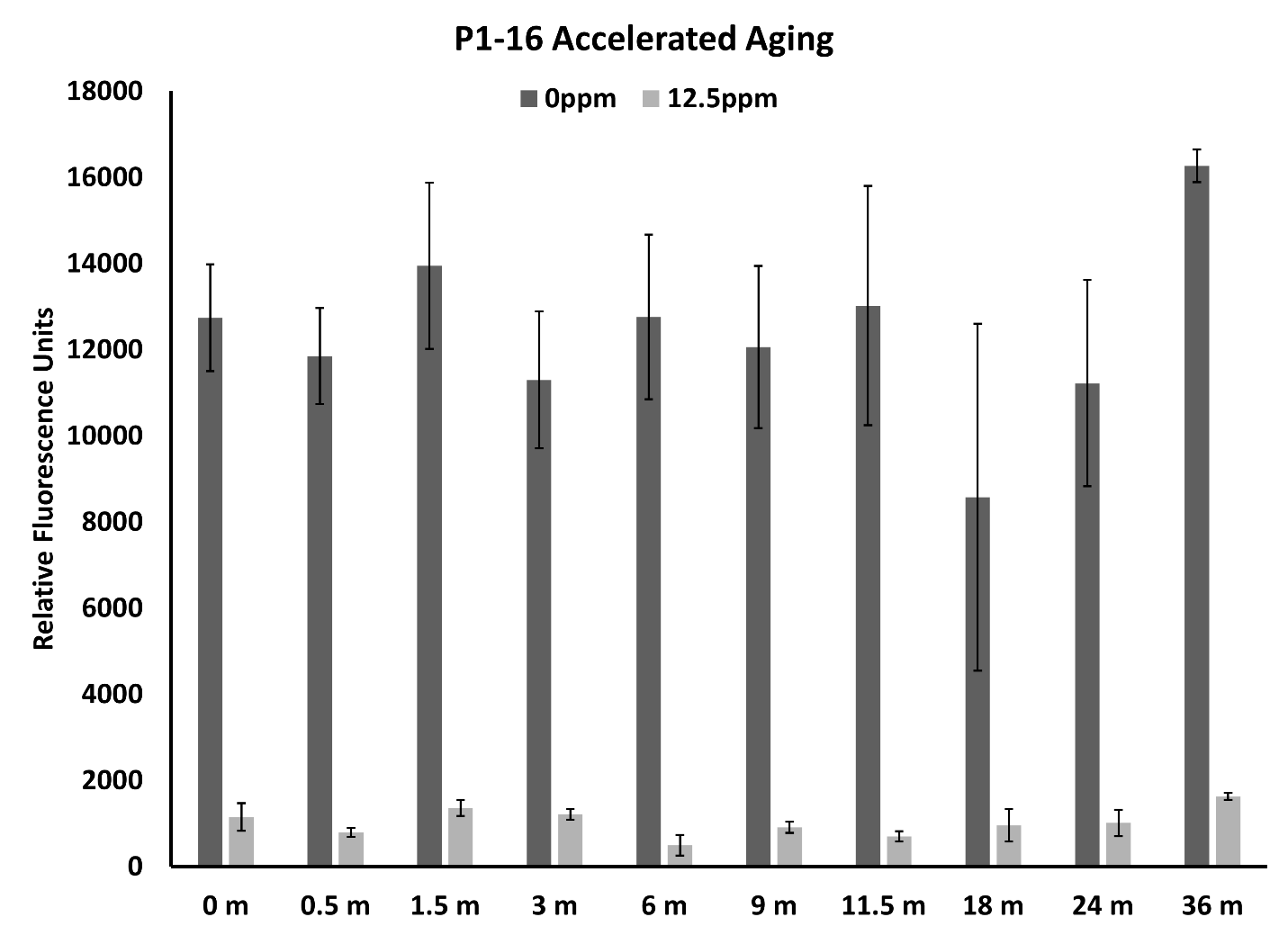


SI Figure 5. AF647-P1-16 retains its sensitivity to peanut over an accelerated aging of 3 years. Time on x-axis is in months (m).

| **Component/Additive** | **Amount (relative to pure component)** | **Example Food and Amount** |
| --- | --- | --- |
| Sucrose | 100% | Table Sugar (100%) |
| Aspartame | 4% | Equal Packet (4%) |
| Sucralose | 1% | Splenda Packet (1%) |
| Insoluble Fiber | 90% | Corn Starch (90%) |
| Soluble Fiber | 1% | Black Beans (10%) |
| Food Coloring | 1% | Trix Cereal (0.1%) |
| Saturated Fat | 100% | Coconut Oil (92%) |
| Unsaturated Fat | 100% | Olive Oil (86%) |
| Sodium | 2% | Cured ham (1.5%) |
| Magnesium | 1% | Pumpkin seeds (0.5%) |
| White Vinegar | 25% | Traditional vinaigrette (25%) |
| Citric Acid | 1% | Lemon juice (8%) |
| Gallic Acid | 0.1% | Red Wine (0.1%) |
| Alginate | 1% | Restructured meat (1%) |
| Alginate | 0.1% | --- |
| Lecithin | 1% | Egg (1%) |
|  |  |  |

SI Table 2. Food components and additives tested in guard band studies. Rightmost column lists an example food with the highest expected amount of substance tested.

|  | **0ppm** | | | | | **12.5ppm** | | | | | |
| --- | --- | --- | --- | --- | --- | --- | --- | --- | --- | --- | --- |
| **Food** | **Sample Avg.** | **Sample SD** | **Control Avg.** | **Control SD** | **Ratio** | **Sample Avg.** | **Sample SD** | **Control Avg.** | **Control SD** | | **Ratio** |
| Alfredo Sauce | 93.7 | 5.7 | 47.5 | 8.1 | -97% | 30.3 | 3.2 | 29.2 | 2.8 | -4% | |
| Applesauce | 109.0 | 49.6 | 56.5 | 26.5 | -93% | 16.8 | 2.6 | 19.5 | 2.1 | 14% | |
| Asian Dressing | 53.8 | 18.3 | 30.0 | 5.0 | -79% | 15.2 | 2.4 | 17.0 | 2.3 | 10% | |
| Blue Cheese Dressing | 46.5 | 7.6 | 25.9 | 5.5 | -79% | 14.8 | 1.2 | 17.8 | 2.1 | 17% | |
| Fruit punch | 93.5 | 10.7 | 43.0 | 5.6 | -117% | 27.1 | 2.5 | 29.6 | 3.1 | 9% | |
| Oat cereal | 127.6 | 12.6 | 52.3 | 6.5 | -144% | 32.2 | 6.5 | 29.7 | 6.5 | -8% | |
| Chicken Gravy | 72.1 | 8.3 | 38.2 | 6.4 | -89% | 22.9 | 3.3 | 21.9 | 3.8 | -4% | |
| Coconut Milk | 84.7 | 13.9 | 40.6 | 10.3 | -109% | 23.3 | 2.7 | 24.4 | 3.3 | 5% | |
| Coffee Creamer | 93.9 | 3.5 | 38.6 | 3.6 | -143% | 21.6 | 1.2 | 20.3 | 1.7 | -6% | |
| Flavored tortilla chips | 38.1 | 8.7 | 20.9 | 4.9 | -83% | 21.2 | 4.6 | 17.9 | 4.7 | -19% | |
| Fruit flavored chewy candy | 84.7 | 4.7 | 42.6 | 2.9 | -99% | 19.6 | 5.0 | 24.1 | 5.7 | 19% | |
| Fluff | 106.6 | 8.6 | 55.7 | 5.7 | -91% | 22.9 | 1.9 | 26.6 | 2.3 | 14% | |
| French Dressing | 62.0 | 8.0 | 27.9 | 3.4 | -123% | 17.6 | 4.3 | 18.6 | 5.3 | 5% | |
| Corn chips | 109.5 | 16.8 | 54.5 | 10.2 | -101% | 31.0 | 2.0 | 34.3 | 1.7 | 9% | |
| Sweetened cereal A | 81.7 | 14.6 | 47.5 | 12.6 | -72% | 29.6 | 5.8 | 30.6 | 4.2 | 3% | |
| Frosting | 77.3 | 3.5 | 42.2 | 3.4 | -83% | 23.8 | 2.9 | 28.5 | 2.7 | 16% | |
| Sweetened cereal B | 70.3 | 9.9 | 36.7 | 4.7 | -91% | 21.2 | 0.3 | 23.8 | 1.9 | 11% | |
| Electrolyte beverage | 92.9 | 10.7 | 41.9 | 5.3 | -122% | 26.2 | 2.0 | 27.6 | 2.4 | 5% | |
| Granola | 65.8 | 20.5 | 38.6 | 11.4 | -71% | 28.1 | 3.0 | 23.2 | 0.5 | -21% | |
| Hoisin Sauce | 45.8 | 12.0 | 18.7 | 5.8 | -145% | 17.6 | 2.6 | 16.2 | 3.2 | -9% | |
| Honey | 91.1 | 14.2 | 34.7 | 6.1 | -163% | 23.5 | 2.7 | 25.1 | 4.7 | 6% | |
| Chocolate-covered wafer | 63.2 | 20.6 | 34.1 | 10.4 | -85% | 21.1 | 4.9 | 18.9 | 4.5 | -11% | |
| Marshmallow cereal | 73.3 | 4.5 | 35.9 | 4.4 | -104% | 23.5 | 2.7 | 22.9 | 2.1 | -2% | |
| Mashed Potatoes | 56.6 | 6.4 | 31.7 | 4.9 | -79% | 23.6 | 2.4 | 19.6 | 2.5 | -21% | |
| Meringue | 93.2 | 5.1 | 44.6 | 4.8 | -109% | 19.4 | 1.2 | 21.4 | 1.5 | 9% | |
| Milk Chocolate | 65.3 | 3.8 | 33.7 | 2.6 | -94% | 27.0 | 0.2 | 21.7 | 1.2 | -24% | |
| Mint Chocolate Chip Ice Cream | 71.0 | 6.3 | 34.2 | 4.8 | -108% | 36.1 | 7.3 | 27.8 | 3.9 | -30% | |
| Muffin | 59.3 | 9.1 | 34.5 | 1.7 | -72% | 23.6 | 11.1 | 25.1 | 9.3 | 6% | |
| Mushroom Soup | 67.0 | 3.8 | 32.8 | 3.0 | -104% | 17.3 | 1.1 | 20.6 | 1.8 | 16% | |
| Nacho Cheese | 82.3 | 6.7 | 43.2 | 3.7 | -90% | 22.9 | 1.2 | 25.7 | 1.0 | 11% | |
| Olive Oil | 83.8 | 20.3 | 37.7 | 9.9 | -122% | 22.7 | 2.6 | 24.7 | 2.0 | 8% | |
| Caramel Chocolate Bar | 82.4 | 5.2 | 35.7 | 1.9 | -131% | 35.0 | 5.8 | 25.6 | 7.6 | -37% | |
| Pasta Sauce | 66.8 | 14.9 | 33.7 | 4.8 | -98% | 18.1 | 3.6 | 18.3 | 4.0 | 1% | |
| Pear Baby Food | 94.7 | 6.9 | 38.1 | 3.7 | -149% | 20.5 | 2.9 | 20.6 | 2.9 | 0% | |
| Rainbow Sherbet | 59.1 | 6.2 | 35.4 | 6.0 | -67% | 32.1 | 5.0 | 24.8 | 3.3 | -29% | |
| Ranch Dressing | 40.4 | 4.1 | 24.9 | 4.5 | -62% | 25.6 | 4.5 | 21.7 | 4.2 | -18% | |
| Rice | 79.5 | 22.2 | 43.8 | 10.4 | -82% | 32.4 | 15.9 | 36.7 | 16.0 | 12% | |
| Rice Noodles | 110.8 | 16.5 | 61.5 | 7.5 | -80% | 27.4 | 1.2 | 32.0 | 1.5 | 14% | |
| Sauerkraut | 77.8 | 8.8 | 38.8 | 6.8 | -101% | 21.8 | 2.5 | 21.7 | 3.8 | 0% | |
| Shortbread Cookie | 104.7 | 3.7 | 40.6 | 3.3 | -158% | 20.1 | 1.3 | 20.2 | 1.1 | 0% | |
| Sweet Chili Sauce | 47.0 | 7.5 | 25.9 | 4.9 | -81% | 14.3 | 1.4 | 15.9 | 1.7 | 10% | |
| Tomato Soup | 61.8 | 2.0 | 34.3 | 3.6 | -80% | 19.4 | 1.7 | 21.2 | 3.1 | 9% | |
| Sweetened Cereal C | 99.0 | 5.7 | 46.2 | 5.8 | -114% | 33.0 | 7.4 | 32.6 | 5.8 | -1% | |
| Chocolate-covered cookie bar | 65.6 | 22.2 | 29.5 | 5.4 | -122% | 24.6 | 8.6 | 21.5 | 5.1 | -14% | |
| Vanilla Blueberry Gelato | 49.1 | 2.9 | 28.2 | 0.9 | -74% | 20.1 | 8.5 | 24.9 | 10.3 | 19% | |
| Vanilla Crispy Squares | 98.0 | 10.1 | 48.2 | 8.9 | -103% | 28.4 | 2.0 | 30.0 | 0.7 | 5% | |
| Vanilla Ice Cream | 73.5 | 6.8 | 34.9 | 4.6 | -110% | 29.6 | 3.0 | 21.3 | 2.2 | -39% | |
| Vanilla Wafer | 75.0 | 2.8 | 41.2 | 6.8 | -82% | 18.7 | 4.8 | 21.1 | 4.2 | 12% | |
| White Chocolate | 76.6 | 2.9 | 39.8 | 4.9 | -93% | 20.2 | 1.0 | 21.1 | 2.4 | 4% | |
| Yogurt | 46.1 | 10.0 | 25.3 | 4.7 | -82% | 20.0 | 3.8 | 15.4 | 2.4 | -30% | |

SI Table 3. Assay can differentiate between foods with and without peanut protein. Fifty commercially available foods, spiked with 0 ppm or 12.5 ppm peanut protein, were tested with AF647-P1-16 aptamer. At least four replicates were run for all foods. Test and control intensities reported as averaged relative fluorescence units. Ratio reported as normalized difference (1 – test/control). All foods except onion powder with 0 ppm peanut protein show a percent decease less than -50%, while all foods with 50 ppm peanut flour show a percent decrease greater than -50%.

| **Food** | **Sample Avg.** | **Sample SD** | **Control Avg.** | **Control SD** | **Ratio** |
| --- | --- | --- | --- | --- | --- |
| Granola Bites | 11.6 | 2.9 | 13.4 | 3.7 | 13% |
| Nut Clusters | 16.1 | 6.3 | 17.7 | 5.4 | 9% |
| Chocolate with Peanuts | 12.5 | 7.9 | 14.8 | 3.6 | 15% |
| PB Sweetened Cereal | 18.8 | 4.9 | 19.0 | 5.3 | 1% |
| PB Oat Cereal | 20.3 | 5.7 | 21.4 | 4.8 | 6% |
| PB Chocolate Chip Cookie | 15.9 | 5.4 | 17.9 | 1.3 | 11% |
| PB Crackers | 17.8 | 5.7 | 19.4 | 7.2 | 8% |
| PB Granola Bar | 13.8 | 4.5 | 15.6 | 2.5 | 12% |
| PB Mugcake | 12.3 | 1.3 | 17.0 | 1.6 | 27% |
| PB Chocolate Sandwich Cookie | 10.7 | 2.3 | 16.1 | 1.3 | 34% |
| PB Wafer | 16.4 | 3.0 | 21.1 | 3.9 | 22% |
| PB Snack Cake | 9.8 | 2.3 | 15.2 | 2.6 | 36% |
| Peanut Butter Dressing | 9.5 | 1.6 | 12.2 | 2.2 | 22% |
| PB Cup | 11.3 | 2.7 | 16.8 | 3.5 | 33% |
| Caramel Chocolate with Peanuts | 11.7 | 2.8 | 13.9 | 3.1 | 16% |
| Thai Peanut Noodles | 12.1 | 6.7 | 15.6 | 5.5 | 22% |
| PB Protein Bar A | 21.3 | 5.8 | 27.0 | 7.6 | 21% |
| PB Protein Bar B | 23.9 | 3.1 | 34.7 | 4.8 | 31% |
| PB Sandwich Cookie | 28.9 | 2.6 | 35.4 | 3.3 | 18% |
| Peanut Butter Sauce | 13.9 | 6.3 | 20.1 | 8.3 | 31% |

SI Table 4. Foods that contain peanut test positive for peanut protein in integrated assay. Twenty commercially available foods that contain peanut were tested with AF647-P1-16 aptamer. At least four replicates were run for all foods. Test and control intensities reported as averaged relative fluorescence units. Ratio reported as normalized difference (1 – test/control).

| **SELEX Round** | **Peanut protein conc.** | **Incubation time** | **% Recovery** |
| --- | --- | --- | --- |
| 1 | 2500 ppm | 45 min | 2% |
| 2 | 2500 ppm | 45 min | 11% |
| 3 | 2500 ppm | 45 min | 27% |
| 4 | 2500 ppm | 45 min | 48% |
| 5 | 250 ppm | 30 min | 41% |
| 6 | 25 ppm | 20 min | 24% |
| 7 | 12.5 ppm | 20 min | 29% |

SI Table 5. SELEX rounds for peanut aptamers.

| **SELEX Round** | **Gluten Concentration** | **Incubation Time** | **% Recovery** |
| --- | --- | --- | --- |
| 1 | 10000 ppm | 30min | 9% |
| 2 | 10000 ppm | 30min | 26% |
| 3 | 10000 ppm | 30min | 87% |
| 4 | 1000 ppm | 1min | 2% |
| 5 | 1000 ppm | 1min | 11% |
| 6 | 100 ppm | 1min | 3% |
| 7 | 100 ppm | 1min | 17% |

SI Table 6. SELEX rounds for gluten aptamers.

|  |  | **Selected Sequences** |
| --- | --- | --- |
| **Family 1** | **Core Domain** | XXX XXX XXX  **TGG ATG GGG G**XX XXX XXX XXX |
| 1 | **P1-10** | **GTC CGC AGC TGG ATG GGG GAG TGT CTG GTT** |
| 2 |  | GGC CGA TC**T GGA TGG GGG** CTC GGG CGA GTC |
| 3 |  | GTC CGA CGA **TGG GTG GGG G**AA TGC GAC GGC |
| 4 |  | GTC CTG ATC **TGG** **GTG** **TGG GGG** TGT GCA GGC |
| 5 |  | GTC ATG CGA **TGG ATG GGG G**CT GGC AGT CGT |
| 6 |  | GGC CTG GTC T**GG ATG GGG G**CG GGT ATA GGC |
| 7 |  | GCC CGA CTC **TGG ATG** **GGG G**AA TGC GCA GTC |
| 8 |  | GGA CGC GGC **TGG ATG GGG G**CT TGC ACT GTC |
| 9 |  | GGC CGT GTC **TGG GTG GGG G**TA TGC ACT GTC |
| 10 |  | GGC GGA CGC **TGG GTG GGG G**CG TTG ACT AGT |
| 11 |  | GGG CGG AC**T GGA TGG GGG** CAT TAC TGC GGC |
| **Family 2** | **Core Domain** | XX XXX XXX XXX XX**T GGA AGC GXX** XXX XXX XXX XXX |
| 12 | **P1-16** | **GGG TCG AGC TGA GTG GAA GCG TTT CTC CGT** |
| 13 |  | GGT CAG AGA TGG ATG **TGG AAG CG**C GAC GTC |
| 14 |  | GTG CGC ACG ATG GAT G**TG GAA GCG** CAG TCT |
| 15 |  | GGC CGA GTC GTC ACT TCA AT**T GGA AGC** **G**GA |
| 16 |  | TGG CCG CAC TCT GGG TGT GG**T GGA AGC G**GC |
| 17 |  | GTA CGC CCG ATG GAT G**TG GAA GC**T ACT GTT |
| 18 |  | GGC GTT CAC TGG GTG **TGG AAG C**TT GCG GTC |
| 19 |  | GGA CGA TCT GGG TG**T GGA AGC** **G**GA TGAG GC |
| 20 |  | GGC CGC GAT GGA TG**T GGA AAC G**TC TAG TCA |
| **Family 3** | **Core Domain** | XXX XXX XXX **TGG** XXX XXX X**TG GTG T**XX XXX |
| 21 | **P2-8** | **GTG CAT CGA TGG CGT ATG CTG GTG ATG TGC** |
| 22 |  | GGC ATG CGA **TGG** ATG **TGG TG**T ACC CAG TCC |
| 23 |  | GCT CCG TAC GA**T GG**C TGT GC**T GGT G**AT GTA |
| 24 |  | GTT CTG AC**T GG**G TG**T GGT G**CT GCA CTG TCA |
| 25 |  | CCG TAG CGA CAT CAA GCG G**TG GTG** TGC GTG |
| 26 |  | GGA CGC AGC TTG CCT AC**T GGT GGT** CAC GTA |
| 27 |  | GTC ATG AAA CGA GCC TAC ACG **TGG TG**C ATG |
| 28 |  | GGC CTC GAT GGA TG**T GGT GGT** GCT GTCA |
| 29 |  | GTG CTG AGC TGG GAG **TGG TGG T**GC TTT GGC |
| 30 |  | GTC TGC CGA TGG ATG **TGG TG**T ACG CAG ACG |
| **Family 4** | **Core Domain** | XXX XXX XXX XXX XXX **GTG GGT GTX** XXX XXX |
| 31 | **P2-18** | **GTT CCG CCG ATG GAT GTG GGT GTA GTT GTC** |
| 32 |  | GGC CTG AC**T** **GGG TGT** **GG**T TAG GAA CGC GTC |
| 33 |  | GTG CGC CGA **TGG GTG** **TG**T GTC GTG GCT GGT |
| 34 |  | GTT CTG AC**T GGG TGT GG**T GCT GCA CTG TCA |
| 35 |  | GTC ATA CGA **TGG GTG TGG** TAT GTG TAC GTA |
| 36 |  | GGC CAT CGA **TGG GTG TGG** CTG TAC TTG ACA |
| 37 |  | GTC ATG CGC **TGG GTG TGG** CCT GTT GTA GGC |
| 38 |  | GGA CGC GA**T GGG** C**GT GGG** TAT GCA CTT GGC |
| **Family 5** | **Core Domain** | XXX XXX XX**C GCC CCT TXX** XXX XXX XXX XXX |
| 39 | **PT-31** | **GGA CGC ATC GCC CCT TCG AGT GGA CAG GTA** |
| 40 |  | GGC CAA TG**C GCC CCT TC**A TGT TGT CGT GTA |

SI Table 7. Top 40 Aptamer sequences from SELEX selection for peanut protein.

|  | **5' & 3' sequences of selected aptamers** | %C | %G |
| --- | --- | --- | --- |
| **3' Library sequence** | **TTG ACT AGT ACA TGA CCA CTT GA** | **33%** | **27%** |
| 3'_V1 | TTG ACT AGC AGA TGA CCA TT | 27% | 27% |
| 3'_V2 | TTC AGG CAG ACA CGC TTA TT | 27% | 20% |
| 3'_V3_C rich | AAC TCC CTC TCT GCG AAT TT | 47% | 13% |
| 3' V4_G rich | AAG TCC GTG TGT GCG AAT TT | 13% | 40% |
| **5' Library sequence** | **TAG GGA AGA GAA GGA CAT ATG AT** | **4%** | **35%** |
| 5'_V1 | TGC GGA AGA CAA GGA GAT ATC CCC | 35% | 22% |
| 5'_V2 | TCG CAC ATT CCG CTT CTA CCG GGG | 39% | 26% |
| 5'_V4_G rich | TAG GGA AGA GAA GGA CAT ATG GGG | 4% | 48% |
| 5'_V3_C rich | TAC CAC ATT CCG CTT CTA CCC CCC | 57% | 4% |

| **SELEX Library:** |
| --- |
| TAG GGA AGA GAA GGA CAT ATG AT - N30 - TTG ACT AGT ACA TGA CCA CTT GA |

SI Table 8. 5' & 3' sequences of selected aptamers. These sequences were added to SELEX selected aptamers to help maintain structure while enabling binding to the chip.
